# The impact of the use of antiepileptic drugs on the growth of children

**DOI:** 10.1186/1471-2431-13-211

**Published:** 2013-12-19

**Authors:** Herng-Sheng Lee, Shih-Yu Wang, Donald M Salter, Chih-Chien Wang, Shyi-Jou Chen, Hueng-Chuen Fan

**Affiliations:** 1 Department of Pathology, Tri-Service General Hospital, National Defense Medical Center, Taipei, Taiwan; 2 Center for Molecular Medicine, MRC IGMM, University of Edinburgh, Edinburgh, Scotland, UK; 3 Department of Orthopaedics, Tri-Service General Hospital, National Defense Medical Center, Taipei, Taiwan; 4 Department of Paediatrics, Tri-Service General Hospital, National Defense Medical Center, Taipei, Taiwan

**Keywords:** Antiepileptic drugs, Valproic acid, Oxcarbazepine, Topiramate, Lamotrigine

## Abstract

**Background:**

This study investigated whether long-term treatment with antiepileptic drugs (AEDs) had negative effects on statural growth and serum calcium levels in children with epilepsy in Taiwan.

**Methods:**

Children with epilepsy treated with one prescription of AEDs (monotherapy) for at least 1 year were selected. The AEDs included valproic acid (VPA; Deparkin) in 27 children (11 boys and 16 girls) aged 4-18 years, oxcarbazepine (Trileptal) in 30 children (15 boys and 15 girls) aged 5-18 years, topiramate (Topamax) in 19 children (10 boys and 9 girls) aged 6-18 years, and lamotrigine (Lamicta) in eight children (5 boys and 3 girls) aged 5-13 years. Patients with a history of febrile convulsions were selected as the controls.

**Results:**

One year of VPA treatment significantly impaired the statural growth of pediatric patients with epilepsy (*p* < 0.005) compared with the control group. The underlying mechanism may have been due to the direct effect of VPA on the proliferation of growth plate chondrocytes rather than alterations of serum calcium.

**Conclusions:**

These results raise serious concerns about the growth of pediatric epilepsy patients who use AEDs, and potentially the need to closely monitor growth in children with epilepsy and adolescents under AED treatment, especially VPA.

## Background

The skeletal system determines a person’s height. Although stiff and unyielding, bone is a living tissue that continuously remodels throughout life. Specialized cells are engaged in the bone remodeling and turnover processes, such as osteoblasts initiating bone formation, osteocytes monitoring bone mechanical stresses, and osteoclasts absorbing bone [1]. The growth plate is at the end of long bones, is made up of chondrocytes at different stages of differentiation, and is divided histologically into three distinct zones: resting, proliferative, and hypertrophic [2]. Longitudinal bone growth is primarily achieved through the action of chondrocytes in the proliferative and proliferative zones of the growth plate [3]. Apart from the effects of circulating systemic and local hormones, calcium and other chemicals, which are mainly provided by bone to maintain the intra-and extracellular mineral pools, can work in cohort with osteoblasts, osteocytes, and extracellular matrix proteins to mineralize osteoid [4]. Calcium is crucial for normal epiphyseal growth plate development, and changes in extracellular calcium modulate the function of chondrocytes [5]. Proliferation of epiphyseal growth plates results from a complex interplay among a net effect of hormones and growth factors, which may directly or indirectly affect the serum levels of calcium and the condition of those cells, leading to final stature.

Epilepsy is a chronic condition characterized by recurrent clinical events or epileptic seizures, which occur in the absence of a metabolic or toxic disease or fever [6]. In addition, the diagnosis of epilepsy can possibly be made after only one epileptic seizure if an “endearing predisposition of the brain to future seizures” exists. The World Health Organization (2001) estimates a prevalence of 0.8% in the general population, and the prevalence in Taiwan has been reported to be 0.28% [7]. Epilepsy often requires long-term antiepileptic drug (AED) therapy. However, prolonged AED administration is associated with a number of problems such as behavioral and psychiatric disorders, metabolic and endocrine disorders, idiosyncratic reactions, and drug interaction effects [8]. Although some studies suggest that patients with epilepsy treated with AEDs have an increased risk of fractures, low bone mineral density (BMD), and abnormalities in bone metabolism, skeletal diseases associated with long-term AED treatment are seriously unrecognized [9,10]. In a survey of >1000 adult and pediatric neurologists designed to assess the awareness of the effects of AED therapy on bone health, only 28% of adult and 41% of pediatric neurologists reported screening their patients for bone diseases [11]. A lack of consensus between physicians concerning the impact of AED therapy on bone may put epilepsy patients at risk, especially children, with regard to bone health or developing bone diseases.

Evidence suggests that patients with epilepsy are predisposed to bone problems and fractures [12]. However, one meta-analysis concluded that the deficit in bone mineral density was too small to explain the increase in the risk of fractures in patients with epilepsy [13]. Bone abnormalities such as short stature, abnormal dentition, rickets, and osteomalacia have been reported to be linked to the use of AEDs [12,14,15]. The mechanisms through which AEDs cause abnormal bone metabolism and increase fractures are not fully understood. Reports have shown that hypocalcemia is an important biochemical abnormality in patients receiving cytochrome P450 enzyme-inducing AEDs, which potentially increase the catabolism of vitamin D to inactive metabolites, leading to reduction of calcium [9,10,16]. However, some non-enzyme-reducing AEDs have also been linked with low bone mass [10,17,18]. A new generation of AEDs, including oxcarbazepine (OXA), topiramate (TPM), and lamotrigine (LTG), have been approved as therapeutic options for epilepsy. However, to date, there is no consensus about the effect on bone metabolism in individuals receiving these AEDs, and no definitive guidelines for evaluation or treatment have yet been determined. Most epileptic patients are diagnosed and treated in childhood and adolescence, and this period is crucial in attaining peak bone mass. Therefore, it is worth investigating whether AEDs affect bone growth in pediatric patients with epilepsy.

The maintenance of growth and bone health is a complex process that can be influenced by the underlying diseases and nutritional status of a patient, but also by chemical factors. If AED treatment is associated with disturbance of statural growth and calcium metabolism, clinical parameters such as serum calcium levels and statural growth may reveal abnormalities after AED therapy in pediatric patients with epilepsy. The aim of this study was to evaluate the effects of AED monotherapy including VPA, OXA, TPM, and LTG on alterations in serum calcium levels and statural growth in drug-naïve, Taiwanese pediatric patients newly diagnosed with epilepsy. To gain further insight into the mechanism of action of AEDs on linear bone growth, we examined the effects of AEDs on cultured growth plate chondrocytes *in vitro* on cell proliferation using a tetrazolium methylthiotetrazole (MTT) assay. Our results showed that, instead of affecting serum calcium levels, VPA may interfere with the proliferation of growth plate chondrocytes in a direct manner and significantly affect the statural growth of children with epilepsy. These results raise serious concerns about the growth of pediatric epilepsy patients who use AEDs, and potentially the need to closely monitor growth in epileptic children and adolescents under AED treatment, especially VPA.

## Methods

### Study subjects

From February 2009 to January 2011, children with newly diagnosed seizures, which were classified according to the report of the International League Against Epilepsy (ILAE) Commission on Classification and Terminology 2005 [19], including generalized, tonic-clonic (ICD-9-CM diagnosis code 345.10), absence (ICD-9-CM diagnosis code 345.0), myoclonic (ICD-9-CM diagnosis code 345.1), clonic (ICD-9-CM diagnosis code 345.1), tonic (ICD-9-CM diagnosis code 345.1), atonic (ICD-9-CM diagnosis code 345.1), and focal (ICD-9-CM diagnosis code 345.5) seizures. The children were attending the pediatric outpatient department, emergency department, or were admitted to the pediatric ward and started on standard recommended doses of valproic acid (VPA; Deparkine solution; Sanofi Winthrop Industrie, Paris, France; starting dose 20 mg/kg/day, maintenance dose 20-40 mg/kg); OXA (Trileptal suspension form; Novartis, Rueil-Malmaison, France; starting dose 5-10 mg/kg/day, maintenance dose 20-40 mg/kg); TPM (Topamax 100 mg tablets, Janssen-Cilag, Baar, Switzerland; starting dose 0.5-1 mg/kg/day, maintenance dose 3-9 mg/kg); or LTG (Lamictal 50 mg tablets, GlaxoSmithKline, Zeist, Netherlands; starting dose 0.5 mg/kg/day, maintenance dose 5-15 mg/kg) for at least 1 year. All children were ambulatory and without any dietary restrictions. The serum levels of patients taking VPA were routinely monitored, and the levels were within the therapeutic range (50-100 μg/mL). Patients were excluded if they had: (1) a history of taking AEDs or other medications that affect bone metabolism (e.g., steroids, diuretics, vitamin D, calcium supplements, bisphosphonates, or calcitonin); (2) any endocrine or medical disorders (e.g., hypothyroidism or renal diseases); (3) a history of nutritional deficiency; (4) limitations in ambulation or daily physical activity; (5) any progressive neurological disorders other than epilepsy; and (6) clinical/biochemical evidence of rickets or growth retardation. All of the children resided in Taipei, were ambulatory, had normal age-appropriate activity, and nutritionally adequate diets. Subjects with a history of simple febrile convulsions (ICD-9-CM diagnosis code 780.31) were selected as the control group. Body height, weight, and body mass index (BMI) were recorded. All patients were followed up every 3-6 months at the pediatric outpatient department.

### Estimation of serum calcium

Five-milliliter venous blood samples were collected from all patients for the measurement of serum total and ionized calcium levels. Cobas c501 (Roche Diagnostics, Mannheim, Germany) and NOVA CCX (NovaBiomedical, Waltman, MA, USA) were used for the measurement of serum total and ionized calcium levels, respectively.

### Consent and ethical approval

The current study was approved by the scientific and ethics committees of Tri-Service General Hospital and National Defense Medical Centre, Taipei, Taiwan (TSGHIRB approval number 100-05-239). All parents, guardians, or legal representatives signed an informed consent form before participation in the study.

### Reagents

Dulbecco’s Modified Eagle’s Medium/Nutrient Mixture F-12 HAM Medium (DMEM/F-12) were purchased from Gibco Life Technologies (Carlsbad, CA, USA). Dimethylsulfoxide (DMSO), fetal bovine serum (FBS), and MTT were purchased from Sigma (St. Louis, MO, USA). All other reagents were purchased from Sigma and were tissue culture grade. The drugs were obtained as described above. In the *in vitro* study, the choice of AED concentration was based on therapeutic plasma concentrations of the respective drug in the patients. The following concentrations were used: VPA, 415 μM (60 μg/mL); OXA, 30 μM (7 μg/mL); TPM, 30 μM (10 μg/mL); LTG, 20 μM (5 μg/mL) [20].

### Cell isolation

Chondrocytes were isolated and cultured as described previously [21]. Male 3-week-old Sprague–Dawley rats (50-60 g each) were obtained from BioLASCO Taiwan (Taipei, Taiwan). All experiments were approved by the local institutional animal care and use committee, Tri-Service General Hospital and National Defense Medical centre, Taipei, Taiwan, ROC (IACUC-12-233). The epiphyseal growth plate of the tibia was separated by cleaning the cartilage plate of muscular tissue, periosteum, and perichondrium. The proximal epiphysis was divided by a transverse cut with a sharp scalpel, and the cartilage plate was separated distally from the calcification zone of the tibial metaphysis. Isolated growth plates were digested with 3 mg/mL collagenase type H (Sigma) for 3 h at 37°CC. After thorough washing, cells were counted using a Neubauer chamber. Cell viability, examined by trypan blue exclusion, was >95%.

### Monolayer cultures

Cell monolayers were cultured in DMEM/F-12 medium supplemented with 10% FBS, 100 IU/mL penicillin (Gibco), and 100 mg/mL streptomycin (Gibco). The cells were grown in 75-cm^2^ plastic culture flasks (Corning, Corning, New York, NY, USA) and incubated at 37°CC until confluence. They were then washed three times with phosphate-buffered saline (PBS), harvested using trypsin-EDTA (Gibco), and subcultured at a 1:3 ratio. Chondrocytes were immunopositive for anti-S100 protein (data not shown). Growth-plate chondrocytes grown to passages 3 and 5 were then plated at 1 × 10^4^ cells/mL into 96-well plates for the MTT assay. The medium with the AEDs was changed daily and cells were collected for assay on Day 5. All cells were maintained in an atmosphere of 5% CO_2_ and 95% air at 37°CC.

### Evaluation of rat chondrocyte proliferation by a MTT assay

Cell viability was determined by measuring the activity of cellular dehydrogenase that could cleave MTT (3-(4,5-dimethylthiazol-2-il)-2,5-diphenyl tetrazolium bromide) (Sigma) in a colorimetric assay as described previously [22]. Activate dehydrogenase reduced MTT in viable cells to form insoluble formazan, which was then dissolved in DMSO and quantified spectrophotometrically at 540 nm. Growth-plate chondrocytes (1 × 10^4^ cells/mL) were seeded into 96-well plates (Corning) in triplicate and kept under 5% CO_2_ at 37°CC. After 24 h incubation, the cell culture medium was replaced daily by one containing fresh complete medium or fresh complete medium with 0.1% DMSO as a vehicle, or fresh complete medium with an AED for 5 days. Two hundred microliters of MTT (0.5 mg/mL) was then added to each well and the mixture was left to incubate for 3 h at 37°CC. The reaction was then stopped by injecting 200 μL DMSO per well. The plates were shaken for 5 min, and then the optical density at 540 nm was determined on a microplate reader (μQuant, BIO-TEK Instruments Inc., Winooski, VT, USA) with KC Junior analysis software, version 1.5 (BIO-TEK Instruments). At least three such experiments were performed for each treatment.

### Statistical analysis

All statistical analyses were performed using SPSS software, version 13.0 (Chicago, IL, USA). Age, sex, weight, BMI, AED, and levels of calcium were expressed as the mean ± standard deviation (SD). Comparisons of the data were conducted by one-way analysis of variance (ANOVA). The Student’s paired *t* test was used to compare serial changes in serum calcium after 1-year treatment with AEDs and the control group. Comparisons of the data from cell proliferation studies were carried out by ANOVA. A *p* value <0.05 was considered statistically significant; *represents *p* < 0.05 and **p < 0.005.

## Results

### Clinical characteristics

The demographic characteristics of the patients are shown in Table 1. There were no significant differences between the control and study patients in age, sex, height, weight, or BMI.

**Table 1:** Clinical parameters of children with epilepsy

	**VPA**	**OXA**	**TPM**	**LTG**	**Control**	***p *** **value**
Subject number	27	30	19	8	30	
Age (mean ± SD)	9.59 ± 3.90	10.43 ± 3.73	9.74 ± 3.28	7.50 ± 3.30	9.10 ± 4.22	0.36
Gender (% Female)	59%	50%	47%	38%	40%	0.64
Height (cm mean ± SD)	129.76 ± 20.00	137.83 ± 20.49	133.79 ± 16.83	123.63 ± 16.95	133.77 ± 23.24	0.4
Weight (kg mean ± SD)	32.26 ± 17.47	37.06 ± 16.29	32.42 ± 14.25	26.81 ± 10.43	33.45 ± 16.60	0.54
BMI (mean ± SD)	18.05 ± 4.19	18.65 ± 3.94	17.28 ± 3.42	17.00 ± 3.09	17.59 ± 4.07	0.69

Age, sex, weight, height, and BMI in epilepsy patients before AED treatment and in the control group.

### Changes in statural growth

**Figure 1:**
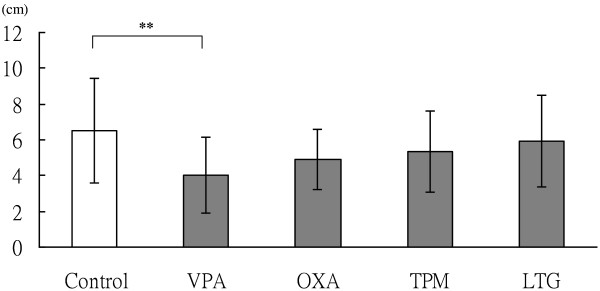
**VPA significantly affected growth of children with epilepsy.** Comparison of changes in body height among the control group and children with epilepsy treated for 1 year with AEDs, including VPA, OXA, TPM, and LTG. The bars represent means, and the whiskers represent standard errors. Significant differences (**, *p* < 0.005) were found between the control group and the group with VPA treatment.

A statistically significantly lower body height was found in patients treated with VPA compared with the controls (*p* < 0.005; Figure 1). However, there were no significant differences between the control group and patients treated with OXA, TPM, or LTG.

### Serum total and ionized calcium levels

**Figure 2:**
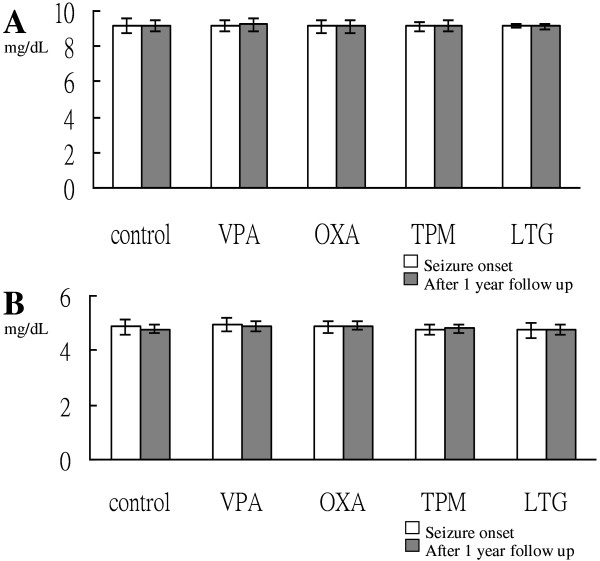
**AEDs did not affect the level of calcium in children with epilepsy.** Comparison in the changes of serum total **(A)** and ionized **(B)** calcium concentration among the control group and children with epilepsy treated for 1 year with AED, including VPA, OXA, TPM, and LTG. The bars represent means, and the whiskers represent standard errors.

Levels of serum total and ionized calcium did not differ significantly among the patients treated with VPA, OXA, TPM, and LTG compared with the controls (*p* > 0.05; Figure 2). None of the drugs affected the level of serum calcium in the epilepsy patients.

### Evaluation of growth-plate chondrocyte proliferation

**Figure 3:**
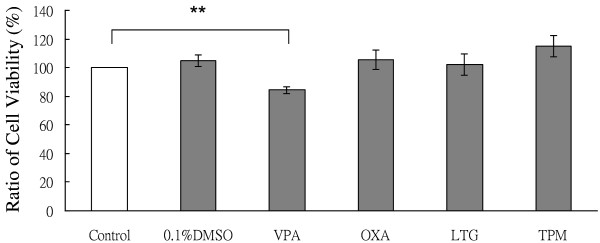
**VPA significantly reduced the proliferation of rat growth-plate chondrocytes.** The influence of vehicle (0.1% DMSO) and AEDs, including VPA, OXA, LEV, LTG, and TPM on chondrocytes of rat growth-plate proliferation in MEM:HAM-F12 (1 : 1) medium with 10% FBS, expressed as cell growth percentages. The mean values are presented on top of the bars with the standard error value. (**, *p* < 0.005).

The influence of the vehicle (0.1% DMSO) and AEDs, including VPA, OXA, LTG, or TPM on growth-plate chondrocyte proliferation was expressed as a percentage of cell growth in six independent experiments. In comparison with the controls, the cell proliferation rate was significantly decreased to 84.45 ± 2.3% when the cells were exposed to VPA. However, there were no significant effects on the proliferation of the chondrocytes with OXA, LTG, or TPM (Figure 3).

## Discussion

In the current study, there were significant reductions in statural growth in the epilepsy patients who were treated with VPA for 1 year compared with the control group. However, there were no significant differences in statural growth in those who were treated with OXA, LTG, or TPM. In support of our findings, Sheth et al. [23] and Kafali et al. [24] reported decreased bone mass in the lumbar spine and middle of the distal radius in children without physical handicaps who were treated with VPA for ≥6 [23] or 18 [24] months. This suggests that VPA can disturb bone growth. Childhood and adolescence are crucial periods in which to attain peak bone mass, and most patients with epilepsy are diagnosed and treated in this period, therefore, AEDs, and especially VPA, should be used with caution in pediatric patients with epilepsy.

Calcium is crucial for normal epiphyseal growth plate development. However, hypocalcemia is reported to affect 3–30% of patients with epilepsy treated with AEDs [25], and this has been postulated to explain AED-associated bone disease. Theoretically, AEDs that induce cytochrome P450 enzymes may cause reduced levels of bioavailable vitamin D, leading to decreased absorption of calcium in the gut, resulting in hypocalcemia and an increase in circulating parathyroid hormone, which then increases the mobilization of bone calcium stores and subsequent bone turnover [9,10,26]. In the current study, no significant changes in serum total and ionized calcium concentrations were found in the patients after 1 year treatment with VPA, OXA, LTG, or TPM. In addition, vitamin D deficiency has been reported to not affect low BMD in epilepsy patients after correcting for age and duration on AEDs [27]. These results and others [28,29] support the notion that AEDs can cause bone loss without inducing hypocalcemia and vitamin D deficiency, suggesting that other mechanisms may be responsible.

VPA, a cytochrome P450 enzyme inhibitor, is widely used for the management of epilepsy [30]. In the current study, the statural growth of pediatric patients was significantly affected by the use of VPA compared with the control subjects, and this was not through alterations in the concentration of calcium. The reported effects of VPA on bone loss in patients with epilepsy are diverse, including accelerated or no bone loss [30-33], hyper- and hypocalcemia [33,34], or normal serum calcium level [35,36]. To clarify these contradictions, we examined the effects of AEDs on the proliferation of cultured growth plate chondrocytes *in vitro*, and showed that cell proliferation was significantly inhibited by VPA, which is similar to our clinical findings. However, also in agreement with our clinical findings, no distinct effects on the inhibition of proliferation in the growth-plate chondrocytes were seen in the patients who were treated with OXA, TPM, or LTG.

OXA, TPM, and LTG are approved for monotherapy or adjunctive therapy in patients with partial and generalized seizures. Despite being safer and having better tolerability, data regarding these new generation AEDs on bone health in children are controversial. OXA and TPM are cytochrome P450 isoenzyme inducers. Epilepsy patients treated with OXA are reported to have an increased risk of fractures [37], lower BMD [28], and decreased 25-hydroxyvitamin D3 levels [38]. TPM is associated with renal calculi, osteomalacia and/or osteoporosis [39], and mild hypocalcemia and increased bone turnover [40]. LTG does not induce or inhibit cytochrome P450 isoenzymes [41]. Children treated with LTG and/or VPA for >2 years have shorter stature, lower BMD, and reduced bone formation compared with controls [15]. However, because of combined therapy, the seizure status in those children may be more severe and their physical activity lower. A lower physical activity may cause more severe bone abnormalities than AEDs do. In fact, all available data indicate that LTG monotherapy does not alter BMD, calcium, or vitamin D levels [16,42,43]. Although we did not find disturbances in serum calcium and statural growth in the epilepsy patients who were treated with OXA, TPM, or LTG, our findings do not contradict previous reports. This is because OXA, TPM, and LTG may alter bone microstructure and bone turnover rate but maintain an adequate bone mass, leading to a normal statural growth rate *in vivo* and a normal proliferation of bone cells *in vitro*. Ultimately, all of these factors may have an impact on longitudinal skeletal growth and risk of fractures.

It was unclear how VPA directly interfered with the proliferation of growth-plate chondrocytes in the current study. VPA at a therapeutic dose is an effective inhibitor of histone deacetylases, producing hyperacetylation of histone tails and chromatin relaxation owing to disruption of histone–DNA and histone–histone interactions [44]. Apoptosis of chondrocytes is the main process for growth-plate remodeling, therefore, it is worth investigating whether VPA delays cell-cycle progression [45], modulates caspases and/or induces apoptosis [46], thereby causing inhibition of cell growth and proliferation, leading to short stature.

The current study had a number of limitations. First, the sample size was small and the duration of follow-up was only 1 year. It is possible that statistically significant lower statures would have been discovered after 1 year in children taking some or all of these AEDs if larger sample sizes and longer duration had been used. Second, the literature shows that enzyme-inducing AEDs increase the catabolism of vitamin D to inactive metabolites, potentially explaining why some enzyme-inducing AEDs are associated with increased risk of osteoporosis [9,14,15]. However, it has been reported that vitamin D deficiency may not affect BMD in epilepsy patients after correcting for age and duration on AEDs [27]. If the level of vitamin D is affected by AED, the downstream of the calcium level should be cascaded. The lack of vitamin D was a limitation of our study for a more comprehensive understanding of AED on growth. Third, rat chondrocytes in the growth plate cannot truly represent *in vivo* human conditions. Finally, this study was not randomized. These limitations may have led to some bias in analyzing the effects of AED on the growth of children with epilepsy.

The use of these AEDs for children and adolescents with epilepsy is growing, and the number of reported side effects of the newer AEDs is increasing. Therefore, our findings are valuable, because we performed a longitudinal study on AED monotherapy that indicated the risks of short stature in pediatric patients receiving AEDs. Early identification and proper management of AED-related growth retardation and associated bone health require greater public awareness and understanding of these adverse effects in children and adolescents.

## Conclusions

AEDs are effective and necessary for children with epilepsy. However, long-term AED therapy, and especially VPA, may predispose patients to growth and bone health abnormalities. Childhood and adolescence are crucial growth periods, thus, prevention of growth retardation and adverse bone health with the use of VPA may be addressed by judicious use of AEDs coupled with improved nutrition and promotion of weight-bearing activities. Moreover, the new generation of AEDs such as OXA, LTG, and TPM may be alternative choices because of fewer adverse effects.

## Competing interests

The authors declare that they have no competing interests.

## Authors’ contributions

HS Lee, DM Salter, and HC Fan were involved in conception and design of the study and drafting the manuscript. HS Lee and DM Salter revised the manuscript critically for important intellectual content. SY Wang, CC Wang, SJ Chen, and HC Fan made substantial contributions to acquisition, analysis, and interpretation of data. All authors read and approved the final manuscript.

## Pre-publication history

The pre-publication history for this paper can be accessed here:

http://www.biomedcentral.com/1471-2431/13/211/prepub
